# Coherent Response of Vietnam and Sumatra-Java Upwellings to Cross-Equatorial Winds

**DOI:** 10.1038/s41598-019-40246-w

**Published:** 2019-03-06

**Authors:** Chau-Ron Wu, Li-Chiao Wang, You-Lin Wang, Yong-Fu Lin, Tzu-Ling Chiang, Yi-Chia Hsin

**Affiliations:** 1 Department of Earth Sciences, National Taiwan Normal University, Taipei, Taiwan; 2 Department of Atmospheric Sciences, National Central University, Taoyuan, Taiwan; 3 Research Center for Environmental Changes, Academia Sinica, Taipei, Taiwan

## Abstract

Upwelling off Vietnam in the South China Sea (SCS) and the Sumatra–Java upwelling in the Indian Ocean significantly modulate regional variation in climate. Although located in different hemispheres, these upwellings nearly concur during the boreal summer; both are the result of wind-induced Ekman divergence. Beyond seasonal time scales, the two upwellings were not synchronous in 1998. In the summer of 1998, upwelling off Vietnam was almost absent, generating the warmest summer on record in the SCS. We demonstrated that the El Niño–Southern Oscillation, which was highly correlated with the upwelling in previous studies, was not solely responsible for this variability. Wind trajectory analyses revealed that cross-equatorial winds, which had passed over the Sumatra–Java upwelling site about 2 weeks earlier, were a rapid force acting on SCS summer upwelling. In the summer of 1998, SCS winds were greatly perturbed due to an anomalous wind path that blew toward the SCS through the Sulu Sea. Our findings suggest that not only the resulting weakening but also the perturbation of the SCS winds prevented the formation of summer upwelling off central Vietnam in that year.

## Introduction

Upwellings are the focus of much research because they pump subsurface nutrient-rich water to the sea surface, which in turn results in significant enhancement of phytoplankton blooms. Seasonal upwelling in the South China Sea (SCS) has been recognized for more than half a century. Wyrtki^1^ showed that upwelling occurs at around 12°N, 110.2°E off Vietnam in the boreal summer during the southwest monsoon, and is accompanied by a > 1 °C drop in sea surface temperature (SST) in June and July. Subsequently, several studies have focused on the impacts of and/or mechanisms underlying this seasonal phenomenon^2–6^. For example, advanced very-high-resolution radiometer (AVHRR) satellite infrared images indicated cold water along the western coast of the SCS and evolved into a cold jet stretching eastward along 11°N–12°N in mid-August 1997^5^. Based on *in situ* measurements and satellite altimeter observations, Hu*, et al*.^3^ examined the vertical structure and physical properties of this cold jet in the southwestern SCS.

Beyond seasonal time scales, summer upwelling and the associated cold SSTs off central Vietnam are also characterized by interannual fluctuations that play a vital role in regional variation in climate. For example, summer upwelling off Vietnam was almost absent in 1998. Consequently, the lack of summer cooling made August 1998 the warmest summer on record in the SCS^7^. The consensus among previous studies is that local time-varying wind forcing was the dominant mechanism for this upwelling. Most of these studies have attributed the interannual variability of the prevailing winds and associated upwelling events to the El Niño–Southern Oscillation (ENSO)^8^, which has the largest impact on interannual variability in the global climate, particularly in the Pacific Ocean. For example, Wang*, et al*.^9^ determined that the mature phase of an El Niño event is usually accompanied by a weakened East Asian winter monsoon. Xie*, et al*.^10^ also related the warm 1998 summer to the ENSO via a high-pressure system in the western Pacific because monsoonal winds in the SCS weakened in the winter of 1997 and persisted into the summer of 1998. However, ENSO may not be solely responsible for the summer upwelling off central Vietnam in the SCS. In terms of its spatial and temporal progression, ENSO matures during the boreal winter and is most active from the central to eastern Pacific. Accordingly, this may not be the complete picture.

This summer upwelling and the accompanying SST fluctuations off central Vietnam play a role on regional climate variations; thus, it is important to elucidate the detailed dynamics of this upwelling. In this study, we investigated how cross-equatorial winds originating from the Indian Ocean may have affected summer upwelling off central Vietnam. We also examined the importance of variation in eastern Indian Ocean upwelling to the climate system of the entire Indian Ocean.

### Variability of summer upwelling

**Figure 1:**
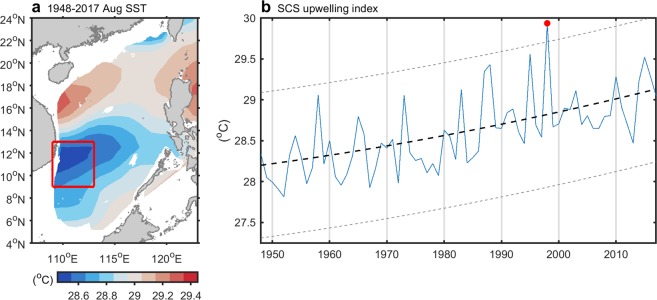
(**a**) The climatological mean sea surface temperature (SST) averaged over 1948–2017 for August. Data over waters shallower than 200 m are ignored. (**b**) Time series of SST for August (blue curve) averaged over the South China Sea upwelling domain (red rectangle in (**a**) 109°E–113°E and 9°N–13°N), together with its residue trend (bold dashed curve), the 99% confidence level (thin dashed curves), and the extreme value of weakened upwelling in August 1998 (red dot).

Before investigating interannual variability in summer upwelling off Vietnam, we examined its spatial variability. Figure 1a shows 70-year (1948–2017) observational SST climatological data obtained from the Hadley Centre Sea Ice and SST dataset (HadISST) for August. SST cooling was centered around 109°E–113°E, 9°N–13°N (referred to as the SCS upwelling domain), spreading slightly toward the northeast over the central SCS. This pattern was consistent with those observed in previous studies in that cold SSTs arose in June and reached maximum magnitude in August, extending eastward over the central SCS^5,10^. This phenomenon explains the robustness of summer upwelling off central Vietnam in this dataset.

Figure 1b shows August SSTs averaged over the upwelling domain during the period 1948–2017, during which time it exhibited significant interannual variation. Ensemble empirical mode decomposition (EEMD)^11^ was performed to extract residual trends. As global warming increases the background SST, the warming signal is evident in the SCS summer upwelling region. SSTs in the upwelling domain increased from 28.2 °C to 29.1 °C during the 70-year period (0.013 °C yr^−1^), which is similar to the global warming rate during the same period (0.01 °C yr^−1^, consistent with study of Stocker*, et al*.^12^). The maximum peak in the summer of 1998 (>99% confidence level) indicates the possible diminishing of upwelling off Vietnam. The lack of upwelling cooling significantly increased water temperatures in the SCS^7,10^. Extended warming had a substantial impact on the hydrography and local climate, and the mechanism responsible for diminishing the upwelling deserves further clarification.

### Cross-equatorial winds

**Figure 2:**
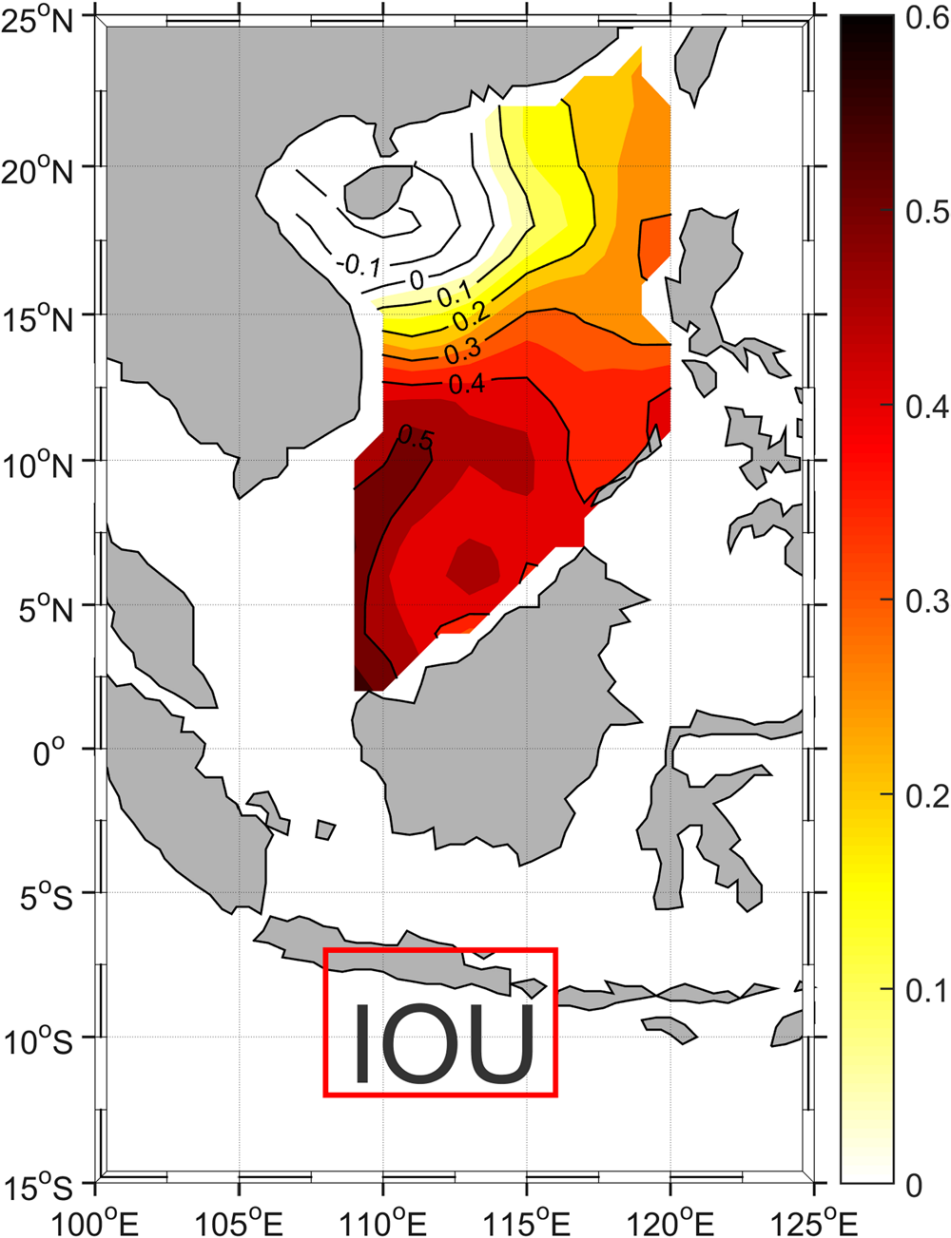
Correlation map between the Indian Ocean Upwelling (IOU, 108°E–116°E, 7°S–12°S) index and the SST anomalies in the SCS with 2-week time lag during the period from 1948 to 2017.

Upwelling is also observed in the equatorial eastern Indian Ocean (Fig. S1), along the coasts of Java and Sumatra. This upwelling, which also takes place during boreal summer, draws much attention because of its critical role for coastal fisheries^13^. To determine whether Indian Ocean upwelling is related to the summer upwelling off central Vietnam in the SCS, a correlation map was used to examine the relationship between SST anomalies in the eastern Indian Ocean in August and those in the SCS (Fig. 2). For simplicity, we quantified SST anomalies averaged in these two areas as the Indian Ocean Upwelling (IOU, 108°E–116°E and 7°S–12°S) and SCS Upwelling (SCSU, 109°E–113°E and 9°N–13°N) indices. The maximum correlation coefficients were distributed northeastward over the SCS, with a pattern corresponding to that of the climatological summer upwelling off central Vietnam (as shown in Fig. 1a). The IOU index in August had a correlation coefficient of 0.41 with the SCSU index in August (above the 99% confidence level) for the period 1948–2017. Sequential upwelling events implied a connection between the two sites. Furthermore, the IOU index was also well (figure not shown) correlated with the cross-equatorial wind stress (averaged over 105°E–108°E, 3°S–6°N), which suggests that the winds were capable of inducing IOU in July/August and then crossed the equator to arrive at the SCS, ultimately giving rise to the SCSU off central Vietnam in August.

**Figure 3:**
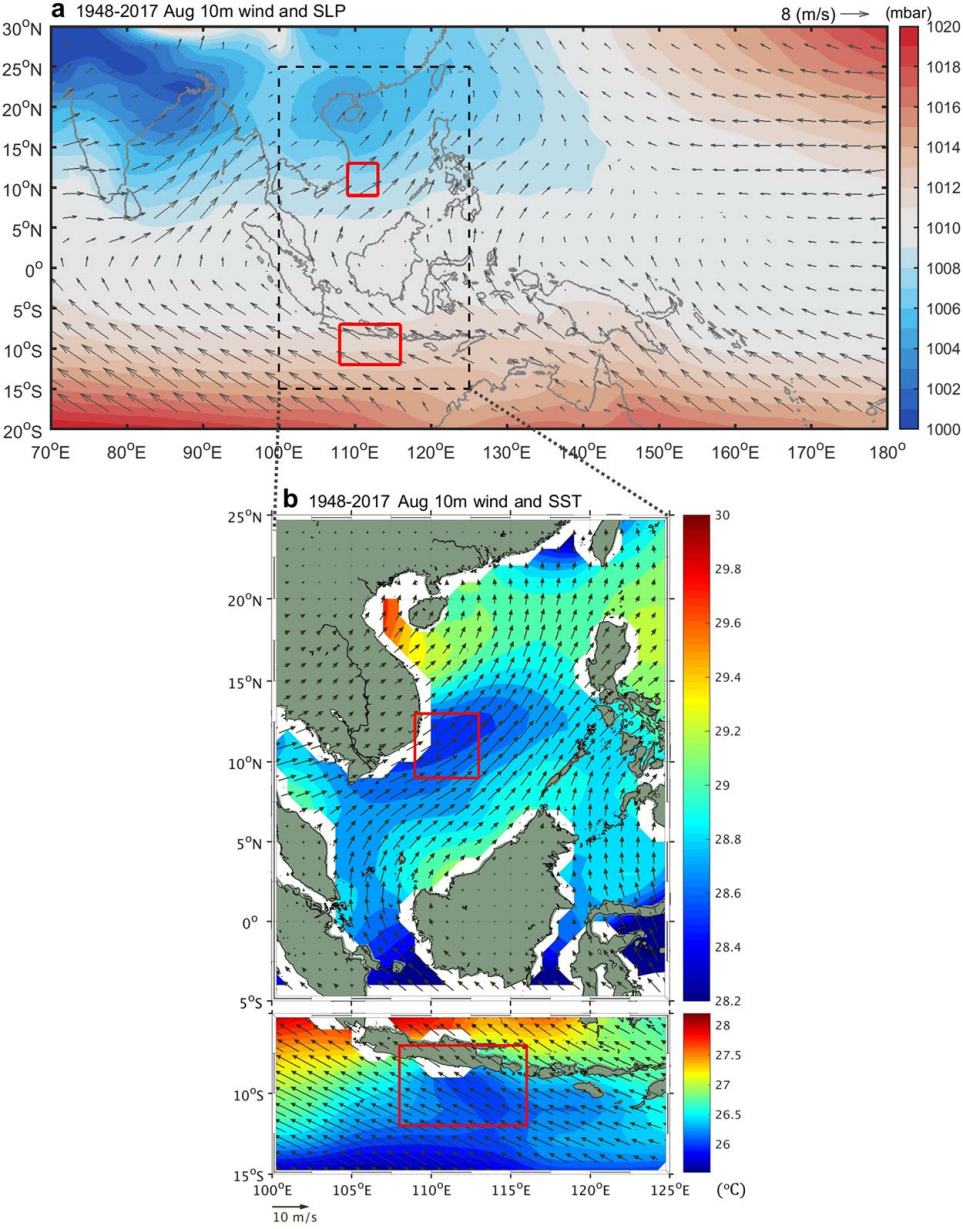
(**a**) The climatological (averaged over 1948–2017) surface winds (vector) and SLP (shading) over the Indo–western Pacific Oceans for August. (**b**) The climatological surface winds (vector) and SST (shading) in the study area for August. In (**a**,**b**), red rectangles identify the SCS upwelling domain (SCSU, 109°E–113°E, 9°N–13°N) and the Indian Ocean upwelling domain (IOU, 108°E–116°E, 7°S–12°S).

Figure 3a shows the climatological distribution of surface wind and sea level pressure (SLP) values from the National Centers for Environmental Prediction (NCEP) for the Indo-western Pacific Oceans for August. Prevailing southwesterly winds in the SCS originated from southeasterly winds in the Indian Ocean off northwestern Australia. The cross-equatorial winds passed over Java, and then crossed the equator and blew in a northeast direction over the entire SCS. Figure 3b shows the relationship between surface winds and the corresponding SST pattern. The cross-equatorial winds apparently drove upwelling in both coastal areas due to Ekman pumping effects off central Vietnam in the SCS and off Sumatra and Java in the Indian Ocean. Variation in boreal summer upwelling in both areas was closely related via cross-equatorial winds blowing from the Indian Ocean to the SCS with the Sumatra–Java upwelling leading the SCS upwelling by one month. The dominant influence of cross-equatorial winds on coastal upwelling has been reported globally, including in the Arabian Sea^14^ and the eastern equatorial Atlantic and Pacific Oceans^15^.

### Wind trajectory analyses

**Figure 4:**
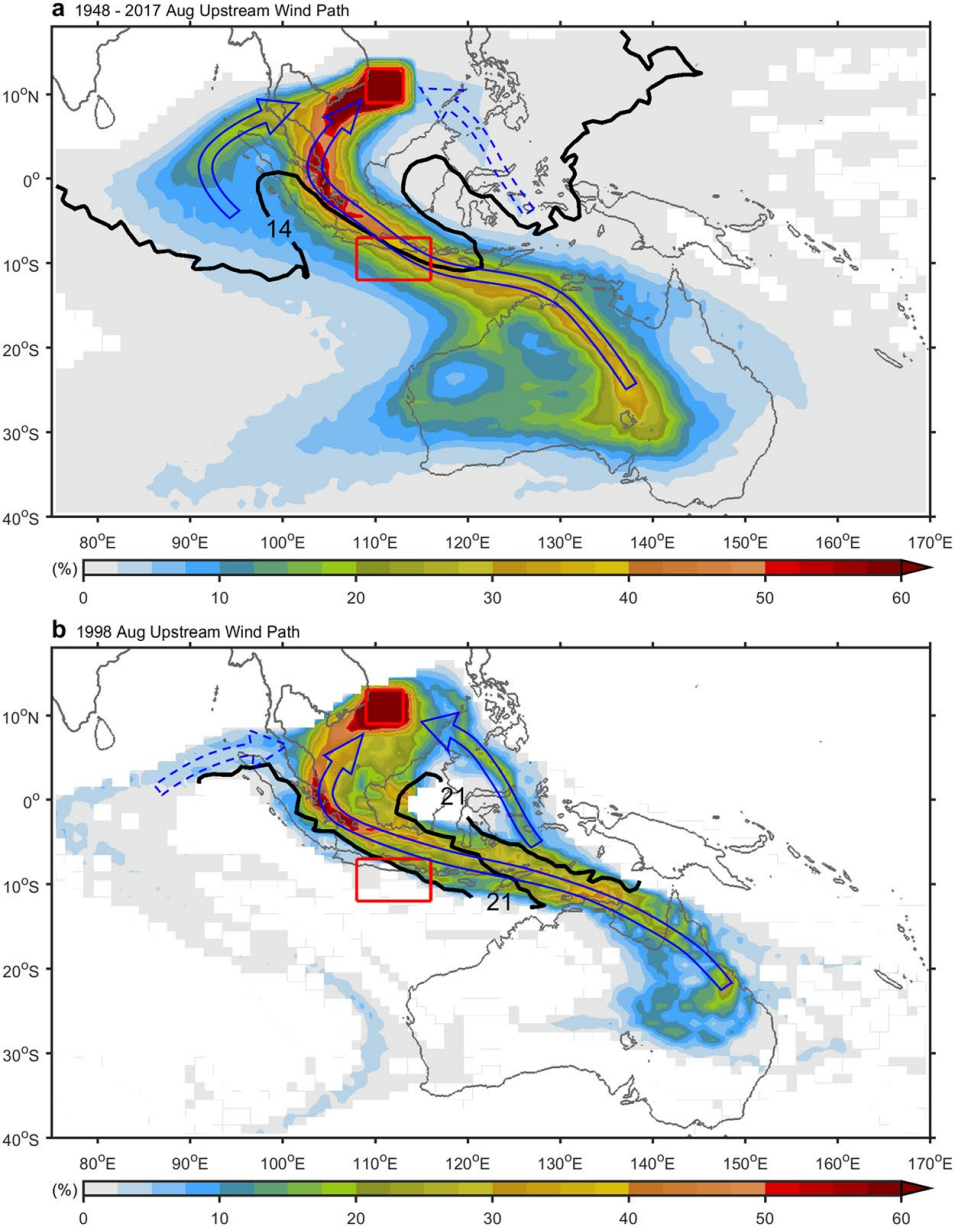
Paths of the cross-equatorial winds. (**a**) Climatological paths of the winds (August 1948–2017). Shading indicates the percentage that particles arrived. The percentage is 100% in the releasing position, the SCSU area. Bold black contour indicates the day when particles arrived (travel time of cross-equatorial winds from the SCSU). (**b**) Same as (**a**), but for August 1998. Red rectangles identify the SCSU and IOU domain.

We conducted a backward-trajectory analysis to better understand the effects of cross-equatorial winds, including their intensity and trajectory, on the summer upwelling off central Vietnam. These analyses were based on a 10 m wind field obtained from the NCEPr1 product for 1948–2017. Air particles were released from locations spread throughout the 109°E–113°E, 9°N–13°N region, with a 1° separation distance in the zonal and meridional directions and one particle released from each location. The release period was set as August 1–31, during the main upwelling episode in the area, and releases were staggered at 1-day intervals. Figure 4a shows two major cross-equatorial wind paths arriving at the SCSU domain in August. In the central path, winds passed through the IOU region (>50% particles) and could be tracked back to their source upstream in central Australia. Connected by cross-equatorial winds, the SCSU lagged behind the IOU (Fig. 4a, bold black contour) by only about 2 weeks. In the western path, winds (~25% particles) originating from the central Indian Ocean looped northeastward into the SCSU area. The time lag of about 2 weeks based on 6-hourly dataset was about the travel time of cross-equatorial winds from the IOU to the SCSU.

Two different wind paths deviated from the 1948–2017 climatological pattern in August 1998 (Fig. 4b). The central path shifted farther east, from 110°E to 120°E, a deviation of approximately 10° from that shown in Fig. 4a. Cross-equatorial winds originating from northeast Australia blew directly northwestward, bypassing the IOU area, and crossed the equator toward the SCSU area. This journey took about 21 days to complete, which indicates that the strength of the wind on the central path was weakened in August 1998 compared to the other years (Fig. 4a). The western path from the Indian Ocean became less significant as the particle percentage decreased from 25% to 5% compared to that shown in Fig. 4a. The eastern path shown in Fig. 4b, which blew to the SCS through the Sulu Sea, exhibited a five-fold increase, from 3.5% to 17.5%.

**Figure 5:**
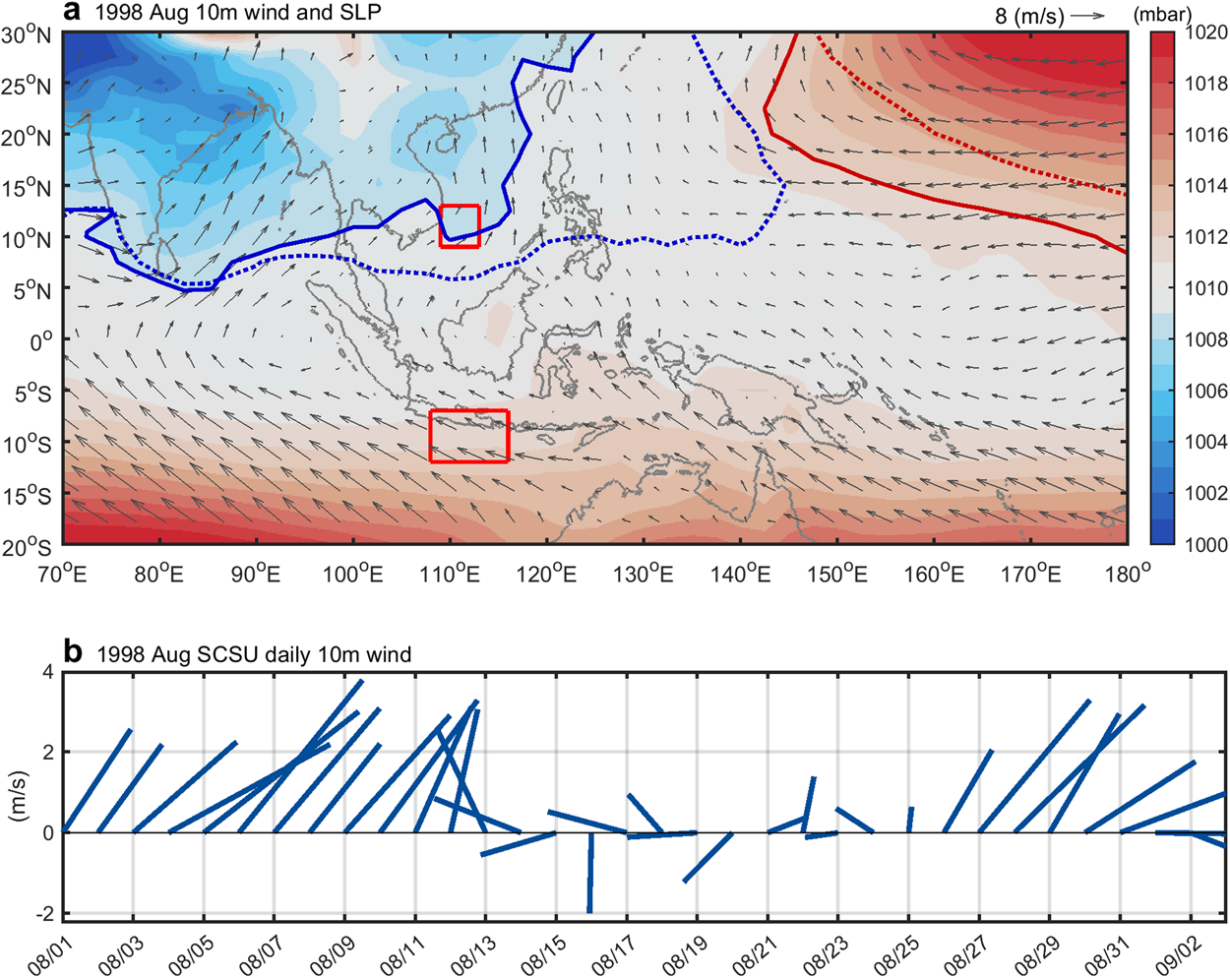
(**a**) Surface winds (vector) and SLP (shading) over the Indo-western Pacific Oceans for August 1998. The western boundary of the North Pacific Subtropical High (red solid and dashed curves indicate 1012 mbar SLP in August 1998 and the climatological August, respectively), and the low pressure boundary in Asia (blue solid and dashed curves indicate 1009 mbar SLP in August 1998 and the climatological August) are also shown. Red rectangles identify the SCSU and IOU domain. (**b**) The daily wind for August 1998 in the SCSU region.

Figure 5a shows the surface winds and SLP over the Indo-western Pacific Oceans in August 1998. Compared to the August climatological patterns shown in Fig. 3a, the North Pacific Subtropical High shifted westward (Fig. 5a, bold red curve) and the SLP in southern China weakened, turning the prevailing winds in the northern SCS northward. Wind intensity also significantly decreased in the SCS basin. The weakening of prevailing southwesterly winds observed in the summer of 1998 was responsible for diminishing the summer SCSU in that year, according to several studies^10,16^. We also found that the winds became much more perturbed in the SCSU area in August 1998 (Fig. 5b), which was unfavorable for the formation of upwelling off Vietnam. This perturbation may be attributed to the enhancement of the eastern path, which blew westward over the SCSU region after leaving the Sulu Sea. The winds frequently encountered each other, causing greater perturbation, as observed in the summer of 1998.

## Discussion

As a result of the close relationship between the two upwelling sites due to their connection via cross-equatorial winds, upwelling variation at the IOU site led SCSU fluctuation by about 2 weeks during boreal summer. The IOU index was also highly correlated with the Indian Ocean Dipole Mode Index (DMI)^17^ (Fig. S2). Over the entire 70-year period, there was a significantly high correlation (−0.77) between the IOU index and the DMI in August. The correlations indicate that, during boreal summer, variability in the IOU index is a proxy for the DMI in the Indian Ocean, and can be served as a precursor to the SCSU index in the SCS.

**Figure 6:**
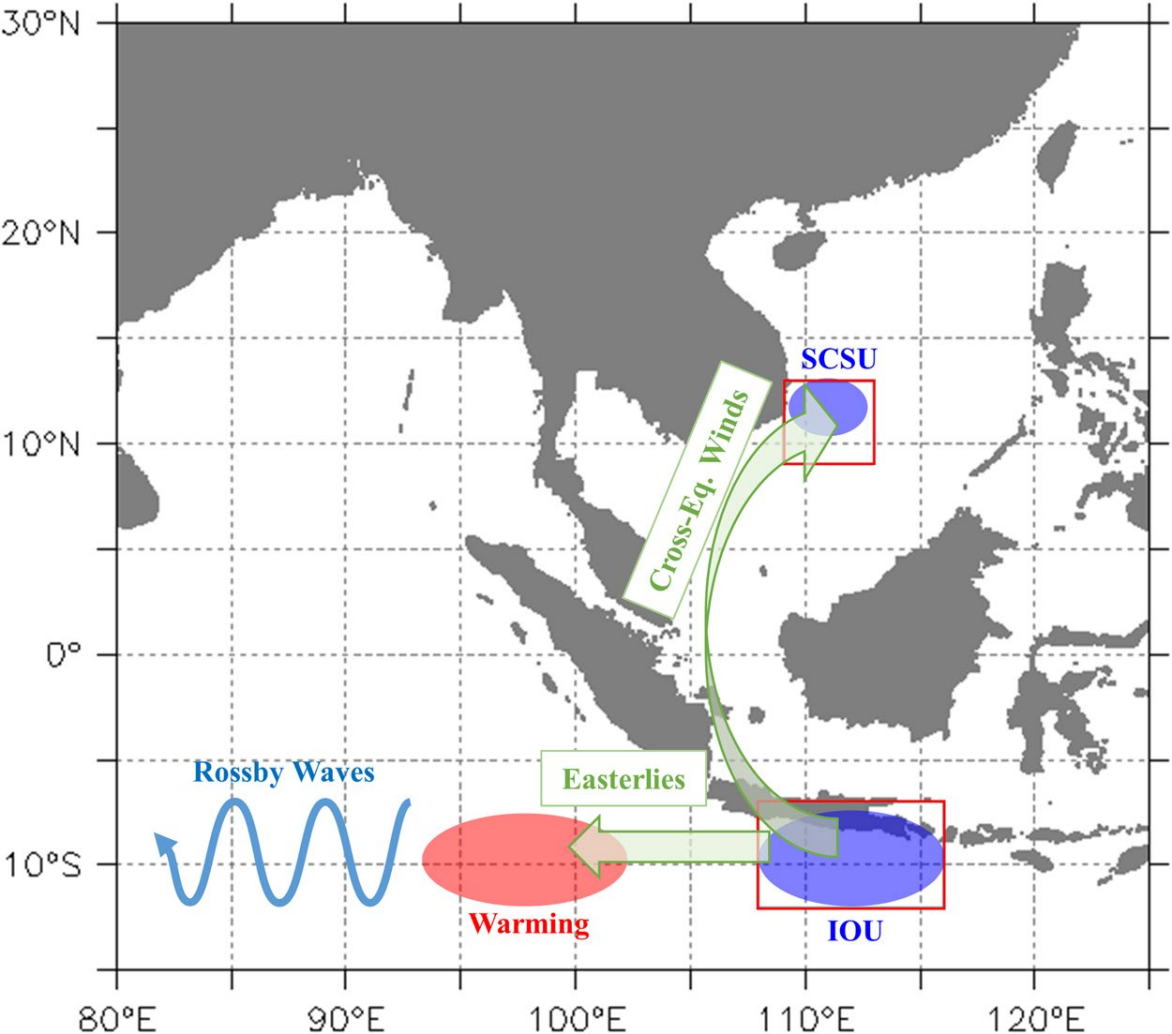
The schematic diagram depicting the sequence among IOU fluctuations and the atmospheric/oceanic variability in the Pacific and Indian Oceans.

Figure 6 shows a schematic representation of the sequence among IOU fluctuations and the atmospheric/oceanic variability in the Pacific and Indian Oceans. The varying IOU has sequences in two directions: northward to the Pacific to influence the SCS and westward to influence the western tropical Indian Ocean. In early boreal summer (July/August), cold SST anomalies first occurred around the Lombok Strait (~9°S, 116°E), accompanied by southeasterly winds in the southeastern tropical Indian Ocean^17^. The southeasterly winds induced coastal upwelling and enhanced cooling in the IOU region. The SST gradient between the IOU region and the equator further intensified and extended the southeasterly winds to the equatorial region. The seasonal difference between the two hemispheres (boreal summer, austral winter) generated a cross-equatorial SST gradient in the equatorial Java Sea and guided the southeasterly winds into southerly winds at 105°E–110°E, blowing toward the SCS. The wind field eventually turned into the prevailing southwest monsoon and induced the SCSU as it approached central Vietnam in August.

The other direction of influence of the IOU was westward, propagating climate variability in the western tropical Indian Ocean. Cold SST anomalies appeared in the Lombok Strait, and the accompanying southeasterly wind anomalies increased the easterly anomalies and slightly weakened the local thermocline. This cooling enhanced and further strengthened the easterly anomalies, triggering westward-propagating downwelling Rossby waves^18^. The deeper thermocline variability and positive SST anomalies induced by the westward-propagating downwelling Rossby waves were closely associated with subsequent warming in the western tropical Indian Ocean.

## Summary

This study demonstrated that ENSO might be a factor, but is not chiefly responsible for, interannual variability in summer upwelling off central Vietnam in the SCS. Cross-equatorial winds originating from the Indian Ocean off northwest Australia were closer and more rapid, acting as a direct force on SCS summer upwelling. Based on wind trajectory analyses for the period 1948–2017, we detected two major cross-equatorial wind paths arriving in the SCSU domain in August. The central path passed through the IOU region and the western path originated from the central Indian Ocean. In the summer of 1998, the western path was replaced by the eastern path, which blew to the SCS through the Sulu Sea. SCS winds became much more perturbed due to enhancement of the eastern path, also resulting in a diminishing of the summer SCSU in 1998. Connected by the cross-equatorial winds, the IOU and SCSU indices were closely correlated, with the IOU index leading the SCSU index by about 2 weeks during boreal summer, indicating variability in the IOU index was a precursor of the SCSU index.

## Methods

### Data sets

Observational SST data used in this study are monthly 1° × 1° Hadley Centre Sea Ice and Sea Surface Temperature dataset (HadISST)^19^, distributed by the Hadley Centre/Met Office (http://www.metoffice.gov.uk/hadobs/). We adopt the 70-year data between 1948 and 2017 for analyses. The monthly 10 m surface wind information, and SLP data are obtained from monthly 2.5° × 2.5° National Centers for Environmental Prediction/National Center for Atmospheric Research (NCEP/NCAR) Reanalysis^20^ (NCEPr1), and interpolated onto a 1° × 1° grid. The daily NCEPr1 10 m winds were especially used for wind trajectory analyses.

### Climate index

The Dipole Mode Index (DMI) is defined as the SST anomaly difference between the western equatorial Indian Ocean (50°E–70°E, 10°S–10°N) and the southeastern equatorial Indian Ocean (90°E–110°E, 10°S–Equator)^17^.

## Supplementary information

Supplementary Info
