# Supplementary material for: Coherent Response of Vietnam and Sumatra-Java Upwellings to Cross-Equatorial Winds
Source: Sci Rep. 2019 Mar 6;9:3650. doi: 10.1038/s41598-019-40246-w (PMC6403322; doi:10.1038/s41598-019-40246-w)
Supplement: Supplementary file 1 — Supplementary Info [file 41598_2019_40246_MOESM1_ESM.docx]

**Supplementary Information**

**Title: Coherent Response of Vietnam and Sumatra-Java Upwellings to**

**Cross‐Equatorial Winds**

Authors: Chau-Ron Wu^1,*^, Li-Chiao Wang^2^, You-Lin Wang^1^, Yong-Fu Lin^1^, Tzu-Ling Chiang^1^, and Yi-Chia Hsin^3^

*^1^Department of Earth Sciences, National Taiwan Normal University, Taipei, Taiwan*

*2Department of Atmospheric Sciences, National Central University, Taoyuan, Taiwan*

*^3^Research Center for Environmental Changes, Academia Sinica, Taipei, Taiwan*

*^*^Correspondence to* [*cwu@ntnu.edu.tw*](mailto:cwu@ntnu.edu.tw)


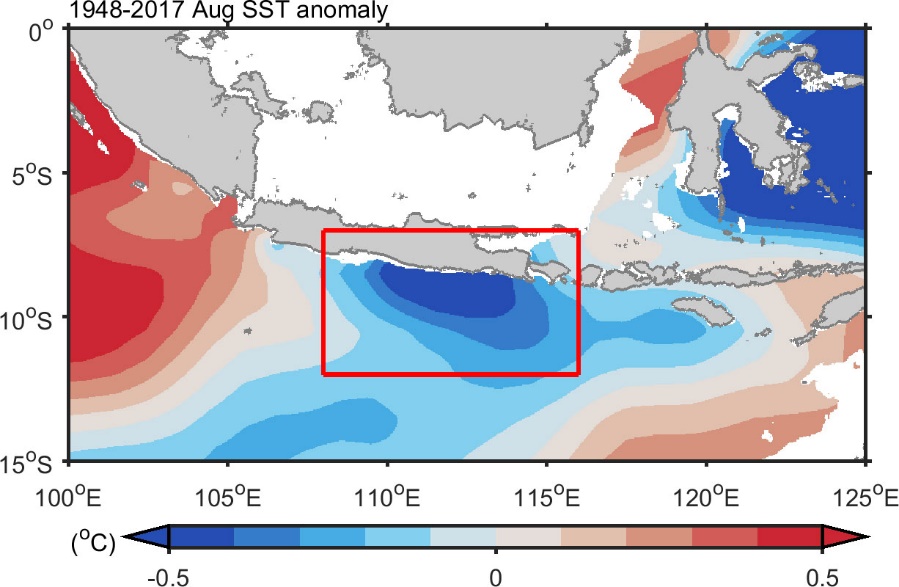


**Fig. S1.** The Indian Ocean Upwelling index (red rectangle; 108^o^E–116^o^E, 7^o^S–12^o^S). Shading indicates the climatological SST anomaly (averaged over 1948–2017) for August, with the zonal average SST from 100^o^E to 125^o^E was removed.

**
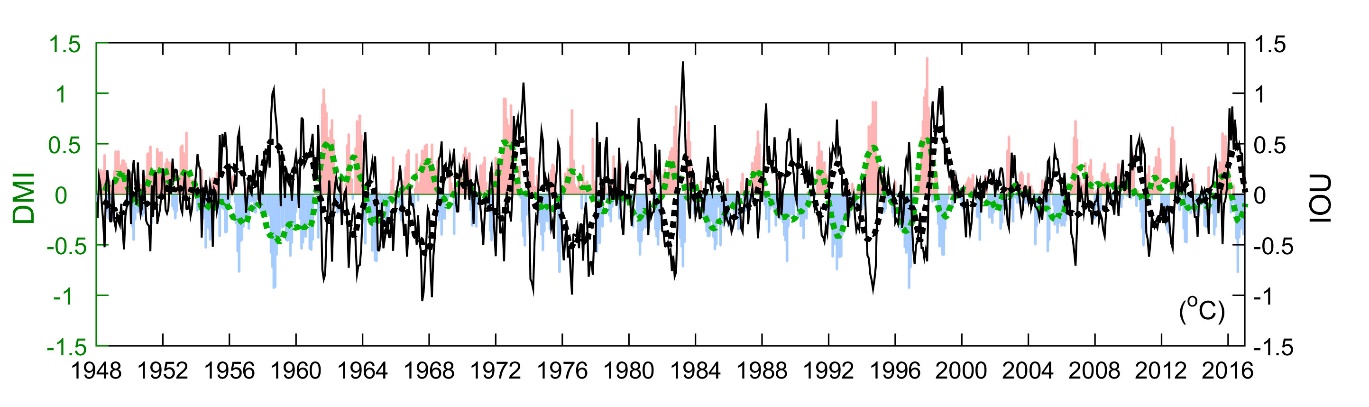
**

**Fig. S2.** Black curve indicates time series of the IOU index, together with monthly Dipole Mode Index (DMI) with positive (negative) values are shown in red (blue) colored bars. Black and green dashed curves are the low-pass filtered (13-month running average) IOU index and DMI, respectively.
